# Supplementary material for: A patient-centred care model for patients with complicated multimorbidity: Protocol for a pilot cluster randomised trial in general practice, municipalities, and hospitals
Source: PLoS One. 2024 Dec 4;19(12):e0310697. doi: 10.1371/journal.pone.0310697 (PMC11616888; doi:10.1371/journal.pone.0310697)
Supplement: S1 Protocol — (PDF) [file pone.0310697.s002.pdf]

**Official title:**

A new care model for patients with complicated multimorbidity.
A cluster-randomised pilot study in general practice, municipalities, and hospitals

**NCT number:**

05406193

**Document date:**

May 23, 2022

## Background

The increasing prevalence of multimorbidity, defined as the coexistence of two or more chronic conditions in the same person [1], [2], is driven by the aging population and improved health technologies and represents a major challenge for healthcare systems. In the Capital Region of Denmark and Region Zealand, about 22% and 37% of the citizens aged 16 years and older suffer from multimorbidity [3], [4]. Around 10% of people with multimorbidity have complicated multimorbidity characterized by suffering from a symptom complex with several concomitant chronic conditions [5]. Researchers have proposed different definitions of complicated multimorbidity such as the severity of conditions, perception of illness, and more [6], [7]. Patients with complicated multimorbidity often have reduced health-related quality of life [8], [9], high treatment burden, polypharmacy, reduced ability to work, low employability, and increased mortality [5].

Healthcare systems are designed to take care of patients with single conditions. Unfortunately, the organisation according to specialities causes problems for the provision of integrated care for patients with multimorbidity. Thus, care pathways are often fragmented, with multiple appointments, more frequent hospital admissions, ambulatory visits, and use of other healthcare. The underlying causes for fragmented care are associated with the lack of a clear division of responsibilities and insufficient coordination between healthcare organisations. This is aggravated by non-integrated economic incentives between general practice, municipalities, and hospitals as well as by IT systems that are not always compatible and different care- and leadership cultures. Disease management programs (DMPs) have been developed to support integrated care but these may not be adequate for patients with multiple conditions and lowered self-care ability, and adhering to multiple DMPs may result in too much medication, conflicting treatments, an overload of appointments, and fragmented health care [10]. Also, providing care to patients with multimorbidity in practices where the rate of patients with multimorbidity is high seems to increase risk of burnout among general practitioners (GPs) [11].

However, while the challenges are widely acknowledged [12] and there is some consensus about key components to improve care [13], knowledge of the most appropriate organisation of healthcare services to ensure patient-centred, high-quality integrated care for patients living with complicated multimorbidity is limited [14], [15].

In Denmark, general practice is the key organisational setting in terms of offering people with complicated multimorbidity integrated, patient-centred care. General practice has responsibility for the continued and longitudinal care of all patients' diseases. The approach is patient-centred and often includes collaboration with the families. A recent study showed that GPs provide chronic care to patients with multimorbidity, and those with low socioeconomic status, but service provision varied highly and more than expected across practices. Remarkably, GPs provided slightly fewer chronic care services than expected in practices where many patients with multimorbidity and low socioeconomic status were clustered, suggesting inverse care law mechanisms [16].

To improve care for patients with complicated multimorbidity in general practice, we developed a complex intervention model named, "patient-centred complex intervention in complicated multimorbidity (CIM)" [17]. The model was developed based on evidence from the Chronic Care Model [18], [19], models of care for multimorbidity [14], [15], [20], and results from our own research [3], [4]. Components of the CIM model included training of health professionals, a longer consultation time to facilitate patient-centred care [18], [19], [21] through the "overview consultation", and reduction of control visits if appropriate. Further, integrated care was supported by a care coordinator, and shared patient information (individualized care plan) between organisations. The CIM model was tested in a feasibility study showing promising results regarding the implementation and acceptance of the CIM both by patients and healthcare professionals [17]. Based on the results from the feasibility study, and other national studies, the present project has developed an improved version of the CIM model named

the CIM2 model [17], [22]. The new CIM2 model includes improved training of healthcare professionals, strengthened identification of patients with complicated multimorbidity, adjustment of extended consultation according to the consultation model of DSAM, improved medical treatment, and strengthened integration of care services between healthcare organisations [17] [23].

The results from the first study of the CIM model [17] attracted the attention of policymakers in primary care, and the new collective agreement for general practice includes a plan to perform a large intervention project based on similar ideas and involving 600 GPs (hence labeled MM600 in the following). This has created a new critical potential for scaling up the elements from the CIM2 model at the national level. However, prior to large-scale implementation – and in accordance with the Medical Research Councils guidance on complex interventions [24] – the intervention must be pilot tested at a smaller scale in order to assess its functionality and effectiveness. For this purpose, we have designed the present pilot study named MM14 (since it includes 14 GPs). The MM14 pilot study will develop and strengthen the CIM2 model, and the results will be used to shape the MM600 national project.

**Aim of the pilot study**

The overall aim of this study is to evaluate important aspects of the “Complex intervention for patients with complicated multimorbidity model, version 2” (CIM2 model) in a pilot cluster-randomised controlled trial (RCT) before conducting a larger cluster RCT[25] being part of the research project described in the Agreement between PLO and Danish Regions 2022 (OK22).

The evaluation of the pilot study has three main objectives [26]:

1) To assess the acceptability, workability and value of the model as experienced by health professionals and patients. In particular, this includes:

a) Assessing if and how the model supports patient-centred care as experienced by patients and professionals
b) Assessing if and how the model supports integrated care between general practice, hospital, and municipality as experienced by patients and professionals
c) Identifying important facilitators and barriers for implementing the model in practice.

2) To assess the acceptability and feasibility of the cluster RCT study design and procedures;

3) To assess and qualify the preliminary power calculations of the Patient Assessment of Chronic Illness Care (PACIC) questionnaire statistical estimates for the main RCT cluster study that is planned in the MM600 project.

The results from the pilot study will be used to develop and strengthen the CIM2 model and the design of the MM600 project.

**Timeline**

The feasibility study took place in the years 2017-18 [17]. The pilot study (MM14), takes place in 2022-2023 with a three months inclusion period and 12-month intervention period. The main RCT study (MM600) will take place in 2023-2024.

**Methods and design**

Study principles and study design

The pilot study includes 14 general practice clinics – seven will be allocated to CIM2-group and seven will be allocated to perform usual care. Each practice will include 25 patients with complicated multimorbidity, thus, in total 350 patients. At 6 and 12 month intervention period, the patients' assessment of perceived patient-centred integrated care, health-related quality of life, and treatment burden will be measured in both groups (Figure 1).

Figure 1. The flow diagram illustrates the structure of the RCT pilot "A new care model for patients with complicated multimorbidity – A cluster-randomised pilot study in general practice, municipalities, and hospitals". Patient Assessment of Chronic Illness Care (PACIC), EuroQol-5 Domain (EQ-5D-5L), Multimorbidity Treatment Burden Questionnaire (MTBQ).

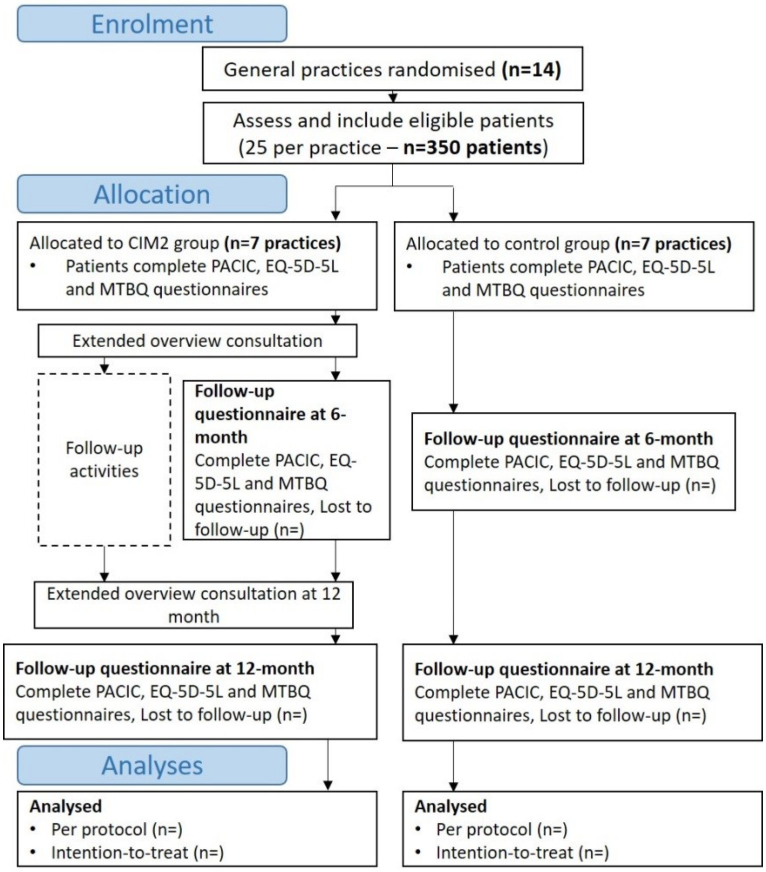


*Setting*

The study will be carried out in Region Zealand and the Capital Region of Denmark, in general practices in three municipalities, in close collaboration with healthcare centers in the municipalities and hospital medical departments hospitals. The general practices should have a minimum of 4,500 patients registered to make sure that the number of patients with complicated multimorbidity reaches the needed number of 25 patients per practice. In addition, the practice needs to be able to include a care coordinator a few hours per week for five months. The municipalities were selected based on the rank of sociodemographic groups (III and IV, lowest) as the prevalence of patients with complicated multimorbidity is higher and the patients are sicker from their multimorbidity. Moreover, the possible benefits of the CIM2 model are expected to be larger in patients from municipalities of these sociodemographic groups.

*Randomization and blinding*

General practices in the designated municipalities are contacted with an invitation to participate in the study. If a general practice agrees to participate in the project, they will receive detailed study information. The 14 participating general practices will be randomised into either the intervention group providing care as described in the CIM2 model or the control group, providing usual care. The general practices will be randomly allocated, at an allocation rate of 1:1, by a computer program. To ensure concealment of allocation, a data manager from another organisation with no interest in the project will provide the information of the randomization to the general practice draw and will be responsible for a randomization list, which will be available to the investigator. Due to the nature of the study, the general practices and the patients cannot be blinded.

*Eligible patients*

The healthcare professionals in general practice identify eligible patients for the project. Identified patients receive oral and written information about the study from the care coordinator. Patients who agree to participate provide informed consent for participation in the study collected by the care coordinator.

Inclusion criteria:

The study group is patients with complicated multimorbidity in general practice that fulfil the fourinclusion criteria:

1. Has more than one of the 3 common chronic diseases (diabetes, chronic obstructive pulmonary, chronic heart conditions) [27].
2. Has been hospitalised, or visited an outpatient clinic due to their chronic diseases during the previous year [17].
3. Take at least five different prescription drugs assessed from the Shared Medicine Card recording (FMK, in Danish “Fælles Medicinkort”) in the general practice [28].
4. The GP or the nurse in the practise recognise the patient as a “demanding” patient with complicated multimorbidity that will benefit from an overview consultation.

Exclusion criteria:

Patients who cannot speak Danish, cannot give informed consent, for example, people with dementia, or who have a life expectancy of less than 12 months will be excluded.

Definition of complicated multimorbidity:

The patient has more than one of the 3 common chronic diseases (diabetes, chronic obstructive pulmonary, chronic heart conditions) [27], has been hospitalised, or visited an outpatient clinic due to their chronic diseases during the previous year [17], take at least five different prescription drugs assessed from the Shared Medicine Card recording (FMK, in Danish “Fælles Medicinkort”) in the general practice [28]. Lastly, some multimorbidity patients are more loosely defined by the GP or the nurse in the practice that recognise the patient as a “demanding” patient with complicated multimorbidity that will benefit from an overview consultation.

## Intervention

Development of the CIM2 intervention

The intervention elements in the CIM2 model have been inspired by the recommendations from the Medical Research Council guidance as described in the following four bullets [25] [24].

Medical Research Council guidance for development of a complex intervention

- Identify and development of intervention: The project has undertaken a literature review in the field of organisation of care in multimorbidity to support the development of the intervention planned to be published in the autumn of 2021. The underlying development theory relies on the Chronic Care Model and uses methods as described for developing and evaluating complex interventions [25]. The interventions will be further developed based on information from participating researchers and health professionals.
- Assessment of the feasibility study and interventions: Assessment of the feasibility study and interventions has been reported previously [17].
- Implementation: the intervention is developed to impact everyday work in the general practice, in the municipality, and hospitals to a minimum. The GPs adopt a new type of consultation for a patient with complicated multimorbidity and both general practice, municipalities, and hospitals are part of the improved integrated care provision. Healthcare services and rehabilitation offered will remain within the already exciting guidelines. The GPs and hospitals are offered the opportunity for using cross-sectorial video conferences as described in the OK22 Agreement.
- Assessment of the complex intervention: This is described in the section "Evaluation" on page 4 in the project description.

The intervention elements in the CIM2 model (Figure 2.)

- Training of healthcare professionals during a four-hour one-day course

The training program is developed in collaboration with The Danish College of General Practitioners. Healthcare professionals from general practice, nurses and physiotherapists from the municipalities, and healthcare professionals from the out-patient clinics participate in a training program. Healthcare professionals from general practice will receive training in project content, and methods to recruit and include patients (including informed consent). Further, does the education program offer training in the collection of patient data comprising the use of REDCap software for patient questionnaires.
For general practices allocated to the intervention group, the training also includes how to provide medical care for patients with complicated multimorbidity, case-based teaching, patient centred methods, structure and implementation of the extended overview consultation, and development of the care plan. Further, the training cover methods for improving collaboration and integration between general practice, the municipality, and the out-patient clinics using methods known from the national disease management programs.

- The extended overview consultation (in Danish "overblikskonsultation") in general practice

The patient intervention starts with an extended overview consultation, lasting 45 minutes, with the GP, the patient (and maybe a relative), and the care coordinator. The consultation is based on the guidance for a patient-centred overview status for patients with multimorbidity published by The Danish College of General Practitioners [29]. The aim is to get an overview of the patient's conditions, problems, and needs. The patient's goals, preferences, and needs are identified, and treatments of the patient's various conditions are prioritized. The GP decides who has the treatment responsibility for each condition. The patient's resources for handling the chronic diseases are elicited, and if the patient is motivated, a referral to a relevant rehabilitation program in the municipality is planned [30]. The individual care plan is developed, covering planned activities in the three sectors that will take place within the 12-month intervention period. The GP will try to reduce the number of outpatient visits in the hospital or replace

outpatient visits with GP visits if the patient and the specialist agree. The GPs are reimbursed with 1.000 DKK. for the extended overview consultations

Figure 2. Central activities during the implementation and intervention phases of the study "Complex intervention for patients with complicated multimorbidity model version 2 (CIM2)" over time.

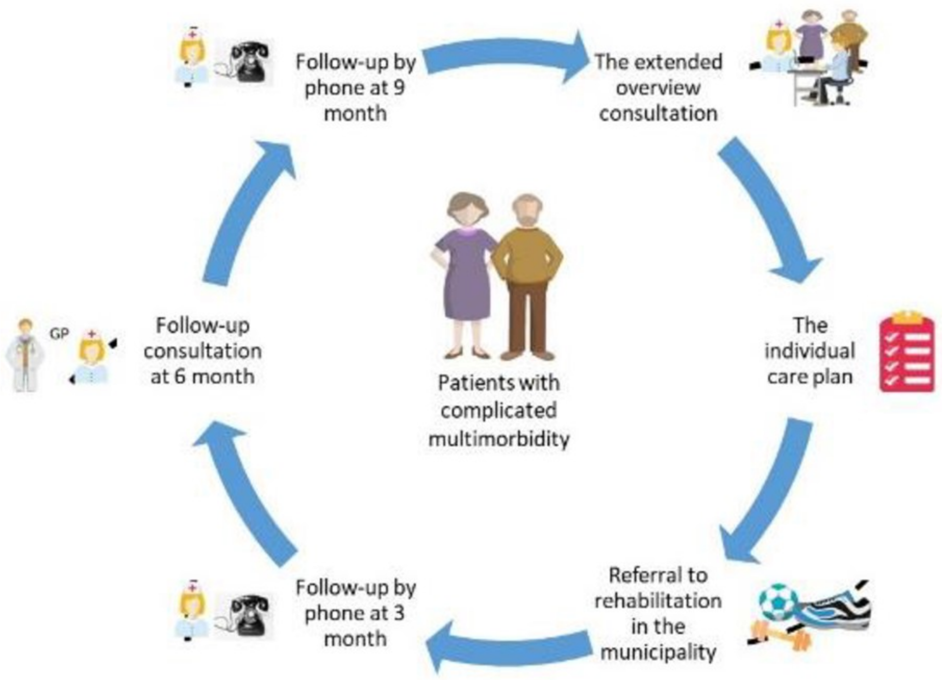


- <u>The individual care plan</u>

The individual care plan includes 6 themes: [29] 1) Listing of important diagnoses, 2) Overview of the patient's prescription drugs, 3) Prioritising the patients' treatment goals (using shared decision-making), 4) Development of a coordinated care plan with telephone follow-up and future visits, 5) Plan for potentially reducing the number of visits to hospital out-patient clinic for example by shifting hospital outpatient clinic visits to general practice, and 6) Referral to community-based rehabilitation. The care plan is printed for the patient.

- <u>Care coordinator</u>

General practice coordinates the planned patient care between general practice, the municipality, and the hospital, and follow-up on the execution of planned healthcare activities. The care coordinator function might be undertaken by the GP or the nurse in the practice. The practice plans the division of responsibilities and tasks in the project between the GP and the nurse.

- <u>Follow-up activities</u>

The follow-up activities include telephone calls to the patient by the care coordinator at relevant time intervals according to the severity of conditions and other important factors to follow up on planned activities with the patient. A second extended overview consultation takes place after 12 months.

- <u>Integration of care elements</u>

The individual care plan is shared electronically with the healthcare center in the municipality and with the outpatient clinics using the standard IT-communication tool provided by MedCom and a routinely used national standard in general practice, hospitals, and municipalities.
The GPs and hospitals are offered the opportunity for using cross-sectorial video conferences to plan the patient pathway as described in the OK22. This means that the GP arranges the conference when there is a professional indication for the conference. The GPs' participation in the videoconference is reimbursed.
The health professionals in the municipality provide information to the GP when patients have finished a rehabilitation program.

*The control group – usual care*
Patients with a GP allocated to the control group will receive usual care. To reduce the possibility of the Hawthorne effect, patients are offered a routine consultation conversation with a focus on patients everyday life with the nurse in general practice.

## <u>**Evaluation and data collection**</u>

The main evaluation objectives of the study will be addressed as follows:

*1) Assessing acceptability, workability and value for health professionals and patients*
This part of the evaluation will investigate how professionals and patients experience the model in terms of acceptability, workability and value. In particular the evaluation will assess whether the intervention supports patient-centred care and integrated care between general practice, hospital, and municipality. Also, this part of the evaluation will identify important facilitators and barriers for implementing the intervention in practice. These objectives will be achieved using a mix of qualitative and quantitative data.
At the end of the intervention, qualitative semi-structured interviews are performed with health professionals and patients. The interviews will include GPs and practice nurses from the seven practices in the intervention group as well as a number of healthcare professionals from the municipalities and hospitals who become involved in the cross-sectorial collaboration with the practices in the intervention group. Additionally, 15 patients will be interviewed.
The interviews will focus on the participants' experiences with the extended overview consultation and with the collaborative cross-sectorial elements of the intervention (especially video conferences and sharing of patient data). The interview guides will be informed by Normalization Process Theory (NPT) – a widely used theory to study the implementation of complex interventions in health care [31]–[33]. Following NPT, the interviews with health professionals will explore how they make sense of the intervention, how they engage with its various elements, how they operationalize the intervention in practice, and how they assess the value of the intervention. The interviews with patients will explore how the patients have experienced their treatment trajectory in terms of coherence and understanding of health care needs in relation to their expectations to integrated care. Also, the interviews with professionals and patients will explore their perceptions of facilitators and barriers to patient-centred and integrated care as well as their suggestions for how care can be improved. The interviews are recorded, transcribed, and subsequently analysed by thematic analysis and NPT.
Further, since the extended overview consultation is a key element in the intervention, video recordings of conducted intervention consultations are gathered and analysed to evaluate if the consultation

supports the relational competencies of the GPs needed to ensure that the patients concerns and priorities are articulated and taken into account. A combination of interactional linguistics [34] and a psychological interpretative framework [35] is used to analyse the relational aspects of the interactions. The video recordings will furthermore be used for evaluation in the conduction of video-stimulated recall interviews (VSRI) [36]. These interviews will elucidate how elements of the model affect the GPs' ability to work with relational competence, to help reduce work burden and prevent burnout. The analysis conducted for this evaluation will consist of Conversation Analysis and a psychological interpretive framework [35].

To supplement the qualitative data on the delivery of the intervention, quantitative data will be generated from the REDCap data collecting program (e.g., number of consultations delivered, number of care plans prepared, and the extent of cross-sectorial communications).

Based on the overall analysis, a proposal is prepared for the possible adjustments of the CIM2 model before the national trial. Subsequently, the results (including any adjustment proposals) are communicated to relevant stakeholders, who are given the opportunity to comment on the results, which may give rise to further adjustment to the CIM2 model before the national trial.

*2) Assessing the acceptability and feasibility of the cluster RCT study design and procedures*

The acceptability and feasibility of the study's tools and procedures for randomisation, recruitment, and data collection will be assessed through qualitative interviews with health professionals and patients supplemented by quantitative data generated by the study (e.g., variations in the number of recruited patients and questionnaire response rates). Patient characteristics according to age, gender, chronic conditions, medication, and care plans will be presented to the general practitioners, nurse care managers, and specialists to validate whether the identified and recruited patients are the "right" patients in need of the CIM2 model and the inclusion criteria are relevant.

Patient-reported questionnaire-data for Patient Assessment of Chronic Illness Care (PACIC), EuroQol-5 Domain (EQ-5D-5L), and Multimorbidity Treatment Burden Questionnaire (MTBQ).will be collected by electronic links by the REDCap software provided to patients at baseline, at 6-month follow-up, and at 12-month follow-up. If patients do not have IT-support at home, they will receive the questionnaires on paper.

The applicability of the following questionnaires will be evaluated from the completion rates of the questionnaires: 1) PACIC [37], [38], 2) EQ-5D-5L, and 3) MTBQ [39].

*3) Assessing and qualifying the preliminary power calculations of the PACIC questionnaire statistical estimates for the main RCT cluster study that is planned in the MM600 study*

Prior to the beginning of the pilot study, we performed a preliminary power calculation to get an understanding of the size of a future main RCT and whether this seemed realistic to use the PACIC questionnaire assessed from the statistical power of the questionnaire. To perform this power calculation, we made several assumptions. First, to our knowledge, no minimal important difference, which provides a "measure of the smallest change in the patient-reported outcome of interest that patients perceive as important", has been defined for the PACIC score. However, previous studies have reported a change in the PACIC score to be 0.36, to be clinically significant [39]. Hence, the power calculations are made towards detecting this difference between the two groups. Second, the power calculations take a potential Intraclass Correlation Coefficient (ICC) of 0.1 into account, which is an upper limit of values found through our literature search for similar studies [39]. Third, literature studies directed a choice of the total variation of 1. Fourth, we require that each clinic will recruit 25 patients and, with a random loss to follow up of 30%. From the stated assumptions, we base ourselves on the following random effects model for those not lost to follow up:

$$Y_i = \alpha_{Group(i)} + Z_{Clinic(i)} + \varepsilon_i, i = 1, \dots, n$$

where the variation of the random effect component $Z$ reflects the ICC. Simulating the above model for 10.000 times per choice of the number of clinics in each group, with individually evaluated random loss to follow up, yields a power of 78%, 84% for 6 and 7 clinics in each group, respectively, based on a two-sided test at the level $\alpha = 0.05$. The uncertainty of these powers is found to be less than 1 percentage point, using the binomial formula. Thus, to obtain a power of 80% for detecting the specified difference $\Delta$ from the random effects modeling, 7 clinics of 25 patients, i.e. 175 patients are needed in the intervention and control group.
The results from the pilot study will strengthen the validity of the estimates and clarify assumptions used in the power calculation. In particular, the results will clarify the need for implementing an ICC in the analysis of the main RCT, and if so, provide the power calculations with an estimated ICC value adapted to local conditions. Furthermore, the results from the pilot study will help to ascertain if 25 patients from each practice is a realistic number to include in the main RCT.

*Data obtained from national health registers*
Data from national and health registers is used for 1) assessment of the comparability of the intervention and control groups according to sociodemographic, chronic conditions, medicine, and 2) assessment of utilization for health-care services.
From registries at Statistic Denmark, data on sociodemographic information in 2021 will be retrieved for both patients in the intervention and control group, using the unique 10-digital personal identification number (CPR number). We will use The Danish Civil Registration System [40] to obtain information on sex, age, and cohabitation. Educational level will be retrieved from The Danish Education Register [41]. Work marked affiliation will be obtained from the Employment Classification Module (AKM) [42], which contains information on economic and employment conditions. Information on utilization of health-care services, such as hospitalizations, bed days, emergency visits, out-patient visits, general practice visits, out-of-hours general practice visits, and specialist visits will be obtained from the Danish National Patient Registry (NPR) [43].

## Dissemination and maintaining of results
The scientific dissemination will consist of the publication of several articles in scientific journals in open-access, high-quality international journals, and a PhD thesis. The results will be presented at national and international conferences as well as at a patient seminar, inviting both patients and relatives included in the study. In addition, the results will be presented to the regional-community coordinating committees in the regions regarding enhancing cross-sectorial collaboration and used to formulate recommendations for organisational cross-sectoral and cross-disciplinary collaboration on complicated multimorbidity – in general practice, municipalities, and hospitals. Furthermore, the newest agreement between the Danish Regions Salary and Rate Board (RTLN) and the Danish Organisation of General Practitioners (PLO) has stated that research results from extended consultations will be used to improve healthcare services for patients with complicated multimorbidity.

## Ethics and approvals
The project was notified to the Danish Data Protection Agency (applied on the 12th of November 2020) and the National Committee on Health Research Ethics in Region Zealand has been notified of the project. According to Danish law, projects like this project do not need ethical approval from a Research Ethical Board (protocol no.: EMN-2020-37129). The project complies with the Declaration of Helsinki. The project is registered at ClinicalTrial.gov.

**References**


[1] M. van Den Akker, F. Buntinx, og J. A. Knottnerus, "Comorbidity or multimorbidity: what's in a name? A review of literature", *Eur. J. Gen. Pract.*, bd. 2, nr. 2, s. 65–70, 1996, doi: 10.3109/13814789609162146.

[2] T. G. Willadsen *m.fl.*, "The role of diseases, risk factors and symptoms in the definition of multimorbidity - a systematic review", *Scand. J. Prim. Health Care*, bd. 34, nr. 2, s. 112–121, 2016, doi: 10.3109/02813432.2016.1153242.

[3] A. L. Blaakilde og B. H. Hansen, "Sundhedsprofil 2017 for Region Sjælland og kommuner – »Hvordan har du det?«", 2018.

[4] Nanna Borup Johansen *m.fl.*, "Sundhedsprofil for Region Hovedstaden og kommuner 2017 – Kronisk sygdom", Center for Klinisk Forskning og Forebyggelse, Bispebjerg og Frederiksberg Hospital, Region Hovedstaden, 2018.

[5] T. Willadsen *m.fl.*, "Multimorbidity and mortality: A 15-year longitudinal registry-based nationwide Danish population study", *J. Comorbidity*, bd. 8, nr. 1, s. 2235042X18804063, 2018, doi: 10.1177/2235042X18804063.

[6] M. Rijken, J. M. Valderas, M. Heins, F. Schellevis, og J. Korevaar, "Identifying high-need patients with multimorbidity from their illness perceptions and personal resources to manage their health and care: A longitudinal study", *BMC Fam. Pract.*, bd. 21, nr. 1, s. 75–75, 2020, doi: 10.1186/s12875-020-01148-3.

[7] M. Heins, J. Korevaar, F. Schellevis, og M. Rijken, "Identifying multimorbid patients with high care needs - A study based on electronic medical record data", *Eur. J. Gen. Pract.*, bd. 26, nr. 1, s. 189–195, 2020, doi: 10.1080/13814788.2020.1854719.

[8] M. Fortin, L. Lapointe, C. Hudon, A. Vanasse, A. Ntetu, og D. Maltais, "Multimorbidity and quality of life in primary care: a systematic review", *Health Qual. Life Outcomes*, bd. 2, nr. 1, s. 51, 2004, doi: 10.1186/1477-7525-2-51.

[9] T. T. Makovski, S. Schmitz, M. P. Zeegers, S. Stranges, og M. van Den Akker, "Multimorbidity and quality of life: Systematic literature review and meta-analysis.", *Ageing Res. Rev.*, bd. 53, s. 100903–100903, 2019, doi: 10.1016/j.arr.2019.04.005.

[10] Mads Aage Toft Kristensen, Tina Drud Due, Bibi Hølge-Hazelton, Ann Dorrit Guassora, og Frans Boch Waldorff, "'More constricting than inspiring' - GPs find chronic care programmes of limited clinical utility. A qualitative study", 2018.

[11] A. F. Pedersen, K. B. Nørøxe, og P. Vedsted, "Influence of patient multimorbidity on GP burnout: a survey and register-based study in Danish general practice", *Br. J. Gen. Pract.*, bd. 70, nr. 691, s. e95–e101, 2020, doi: 10.3399/bjgp20X707837.

[12] J. L. Wolff, "Prevalence, expenditures, and complications of multiple chronic conditions in the elderly.(Archives of Internal Medicine)", *JAMA J. Am. Med. Assoc.*, bd. 289, nr. 7, s. 824-, 2003.

[13] 69 World Health Assembly, "Framework on integrated, people-centred health services: report by the Secretariat", Art. nr. A69/39, 2016, Set: 30. juni 2021. [Online]. Tilgængelig hos: https://apps.who.int/iris/handle/10665/252698

[14] S. M. Smith, E. Wallace, T. O'Dowd, og M. Fortin, "Interventions for improving outcomes in patients with multimorbidity in primary care and community settings.", *Cochrane Database Syst. Rev.*, bd. 3, nr. 3, s. CD006560–CD006560, 2016, doi: 10.1002/14651858.CD006560.pub3.

[15] S. M. Smith, H. Soubhi, M. Fortin, C. Hudon, og T. O'dowd, "Managing patients with multimorbidity: systematic review of interventions in primary care and community settings", *BMJ*, bd. 345, nr. 7874, s. e5205, 2012, doi: 10.1136/bmj.e5205.

[16] A. Prior *m.fl.*, "Chronic care services and variation between Danish general practices: a nationwide cohort study", *Br. J. Gen. Pract.*, s. BJGP.2021.0419-, 2021, doi: 10.3399/BJGP.2021.0419.

[17] H. Birke *m.fl.*, "A complex intervention for multimorbidity in primary care: A feasibility study", *J. Comorbidity*, bd. 10, s. 2235042X20935312, jan. 2020, doi: 10.1177/2235042X20935312.

[18] E. H. Wagner, "Chronic disease management: what will it take to improve care for chronic illness?" 1998.

[19] T. Bodenheimer, E. H. Wagner, og K. Grumbach, “Improving Primary Care for Patients With Chronic Illness: The Chronic Care Model, Part 2”, *JAMA J. Am. Med. Assoc.*, bd. 288, nr. 15, s. 1909–1914, 2002, doi: 10.1001/jama.288.15.1909.
[20] F. R. M. Leijten *m.fl.*, “The SELFIE framework for integrated care for multi-morbidity: Development and description”, *Health Policy*, bd. 122, nr. 1, s. 12–22, 2018, doi: 10.1016/j.healthpol.2017.06.002.
[21] E. M. Langberg, L. Dyhr, og A. S. Davidsen, “Development of the concept of patient-centredness - A systematic review”, *Patient Educ. Couns.*, bd. 102, nr. 7, s. 1228–1236, jul. 2019, doi: 10.1016/j.pec.2019.02.023.
[22] M P Rozing, A Jønsson, R Koster-Rasmussen, T D Due, J Brodersen, og K H Bissenbakker, “The SOFIA pilot trial - a cluster-randomized trial of coordinated, co-produced care to reduce mortality and improve quality of life in people with severe mental illness in the general practice setting”, 2021.
[23] “OK22 | læger.dk”. https://www.laeger.dk/PLO/OK22 (set 10. marts 2022).
[24] K. Skivington *m.fl.*, “A new framework for developing and evaluating complex interventions: update of Medical Research Council guidance”, *BMJ Online*, bd. 374, s. n2061–n2061, 2021, doi: 10.1136/bmj.n2061.
[25] P. Craig, P. Dieppe, S. Macintyre, S. Michie, I. Nazareth, og M. Petticrew, “Developing and evaluating complex interventions: the new Medical Research Council guidance”, *BMJ*, bd. 337, nr. 7676, s. a1655–983, 2008, doi: 10.1136/bmj.a1655.
[26] N. Feeley *m.fl.*, “The importance of piloting an RCT intervention”, *Can. J. Nurs. Res. Rev. Can. Rech. En Sci. Infirm.*, bd. 41, nr. 2, s. 85–99, jun. 2009.
[27] M. L. Schiøtz, A. Stockmarr, D. Høst, C. Glümer, og A. Frølich, “Social disparities in the prevalence of multimorbidity – A register-based population study”, *BMC Public Health*, bd. 17, nr. 1, dec. 2017, doi: 10.1186/s12889-017-4314-8.
[28] S. L. Brilleman og C. Salisbury, “Comparing measures of multimorbidity to predict outcomes in primary care: a cross sectional study”, *Fam. Pract.*, bd. 30, nr. 2, s. 172–178, 2012, doi: 10.1093/fampra/cms060.
[29] “overbliksstatus-og-rsstatus.pdf”. https://vejledninger.dsam.dk/media/files/21/overbliksstatus-og-rsstatus.pdf (set 30. juni 2021).
[30] K. S. Freund, A. D. Guassora, T. Hegelund, L. Hvas, og J. Lous, “Resources in vulnerable young adults: self-assessments during preventive consultation with their general practitioner in Denmark”, *Health Promot. Int.*, bd. 35, nr. 5, s. 1180–1189, okt. 2020, doi: 10.1093/heapro/daz114.
[31] C. May og T. Finch, “Implementing, Embedding, and Integrating Practices: An Outline of Normalization Process Theory”, *Sociology*, bd. 43, nr. 3, s. 535–554, jun. 2009, doi: 10.1177/0038038509103208.
[32] C. R. May *m.fl.*, “Development of a theory of implementation and integration: Normalization Process Theory”, *Implement. Sci. IS*, bd. 4, s. 29, maj 2009, doi: 10.1186/1748-5908-4-29.
[33] R. McEvoy, L. Ballini, S. Maltoni, C. A. O’Donnell, F. S. Mair, og A. Macfarlane, “A qualitative systematic review of studies using the normalization process theory to research implementation processes”, *Implement. Sci. IS*, bd. 9, s. 2, jan. 2014, doi: 10.1186/1748-5908-9-2.
[34] Steensig Jakob, *Sprog i virkeligheden. Bidrag til en interaktionel lingvistik.* Århus: Aarhus Universitetsforlag, 2001. Set: 30. juni 2021. [Online]. Tilgængelig hos: https://www.google.com/search?sxsrf=ALeKk030UNUoyYHowfh5BR_9bcp5qRH4Lg:1625059221119&q=Steensig,Jakob+(2001):+Sprog+i+virkeligheden.+Bidrag+til+en+interaktionel+lingvistik.+%C3%85rhus:+Aarhus+Universitetsforlag.&spell=1&sa=X&ved=2ahUKEwjP9_Osub_xAhXHHXcKHcLQAusQBSgAegQIARAw&biw=873&bih=815
[35] Hansen C., “Relationer i konsultationer: En undersøgelse af relationskompetences anvendelse i almen praksis”, 2021. https://www.academia.edu/s/2776af465e?source=link (set 30. juni 2021).
[36] C. F. Fosgerau og S. Kaae, “Furthering patient-centered counseling: Exploring new aspects around pharmacists’ experiences in pharmacy encounters through video-stimulated recall interviewing”, *Res. Soc. Adm. Pharm. RSAP*, bd. 17, nr. 4, s. 723–732, apr. 2021, doi: 10.1016/j.sapharm.2020.06.018.

[37] H. T. Maindal, I. Sokolowski, og P. Vedsted, “Adaptation, data quality and confirmatory factor analysis of the Danish version of the PACIC questionnaire”, *Eur. J. Public Health*, bd. 22, nr. 1, s. 31–36, feb. 2012, doi: 10.1093/eurpub/ckq188.
[38] E. Glasgow, H. Wagner, D. Schaefer, J. Mahoney, M. Reid, og M. Greene, “Development and Validation of the Patient Assessment of Chronic Illness Care (PACIC)”, *Med. Care*, bd. 43, nr. 5, s. 436–444, 2005, doi: 10.1097/01.mlr.0000160375.47920.8c.
[39] C. Salisbury *m.fl.*, “Management of multimorbidity using a patient-centred care model: a pragmatic cluster-randomised trial of the 3D approach”, *The Lancet*, bd. 392, nr. 10141, s. 41–50, jul. 2018, doi: 10.1016/S0140-6736(18)31308-4.
[40] C. B. Pedersen, “The Danish Civil Registration System”, *Scand. J. Public Health*, bd. 39, nr. 7_suppl, s. 22–25, jul. 2011, doi: 10.1177/1403494810387965.
[41] V. M. Jensen og A. W. Rasmussen, “Danish education registers”, *Scand. J. Public Health*, bd. 39, nr. 7_suppl, s. 91–94, jul. 2011, doi: 10.1177/1403494810394715.
[42] F. Petersson, M. Baadsgaard, og L. C. Thygesen, “Danish registers on personal labour market affiliation”, *Scand. J. Public Health*, bd. 39, nr. 7_suppl, s. 95–98, jul. 2011, doi: 10.1177/1403494811408483.
[43] M. Schmidt, S. A. J. Schmidt, J. L. Sandegaard, V. Ehrenstein, L. Pedersen, og H. T. Sørensen, “The Danish National Patient Registry: a review of content, data quality, and research potential”, *Clin. Epidemiol.*, bd. 7, s. 449–490, 2015, doi: 10.2147/CLEP.S91125.
